# Age as a Predictor of Significant Fibrosis Features in HBeAg-Negative Chronic Hepatitis B Virus Infection with Persistently Normal Alanine Aminotransferase

**DOI:** 10.1371/journal.pone.0123452

**Published:** 2015-04-17

**Authors:** Youwen Tan, Yun Ye, Xinbei Zhou, Li Chen, Danfeng Wen

**Affiliations:** 1 Department of Infectious Diseases, The First Affiliated Hospital of Soochow University, Suzhou, China; 2 Department of Hepatosis, The Third Hospital of Zhenjiang Affiliated Jiangsu University, Zhenjiang, China; Kaohsiung Medical University Hospital, Kaohsiung Medical University, TAIWAN

## Abstract

**Background:**

Although alanine aminotransferase (ALT) levels reflect the degree of liver damage, not all patients with chronic hepatitis B virus (HBV) infection exhibit persistently elevated ALT levels. In the present study, we aimed to comprehensively evaluate the characteristics of histological abnormalities in a large population of Chinese patients with chronic HBV and persistently normal ALT levels.

**Methods:**

In total, 2303 consecutive patients who underwent liver biopsy were screened. Of these patients, 273 were categorized as having persistently normal ALT levels (PNALT), whereas 618 were categorized as having persistently or intermittently elevated ALT levels (PIALT). All these patients had at least three ALT values recorded in the year prior to the baseline liver biopsy.

**Results:**

Significant necroinflammation was observed in 9.7% (11/113) patients with PNALT, 23.3% (42/180) patients with PIALT (ALT 1–2× upper limit of normal [ULN]), and 27.8% (42/151) patients with PIALT (ALT > 2× ULN), whereas significant fibrosis was observed in 8.8% (10/113) patients with PNALT, 27.8% (42/151) patients with PIALT (ALT 1–2× ULN), and 21.2% (32/151) patients with PIALT (ALT > 2× ULN). Multiple logistic regression analysis indicated that age parameters were associated with significant histological abnormalities in patients with PNALT. The area under the curve showed that age was associated with significant fibrosis characteristics in patients with hepatitis B extracellular antigen (HBeAg)-negative PNALT.

**Conclusion:**

Significant histological abnormalities are not often observed in Chinese patients with PNALT. Interestingly, age appears to be a predictor of significant fibrosis in patients with HBeAg-negative PNALT.

## Introduction

Approximately one-third of the world’s population possesses serological evidence of past or present hepatitis B virus (HBV) infection, and 350–400 million people are known to be chronic HBV surface antigen (HBsAg) carriers. The disease spectrum and natural history of chronic HBV infection are diverse and variable; they range from having an inactive carrier status to having progressive chronic hepatitis B (CHB), which may then progress to cirrhosis and hepatocellular carcinoma (HCC)[1, 2]. Chronic HBV infection is a dynamic process, and its natural history was schematically divided into five phases by the European Associated for the Study of the Liver (EASL) Clinical Practice Guidelines (2012)[1]: (1) the “immune tolerant” phase is characterized by hepatitis B extracellular antigen (HBeAg) positivity, high levels of HBV replication (reflected by high levels of serum HBV DNA), normal or low aminotransferase levels, mild or no liver necroinflammation, and no or slow fibrosis progression; (2) the “immune reactive HBeAg-positive phase” is characterized by HBeAg positivity, relatively lower levels of viral replication compared to the immune tolerant phase (as reflected by lower serum HBV DNA levels), increased or fluctuating aminotransferase levels, moderate or severe liver necroinflammation, and more rapid progression of fibrosis compared to the previous phase; (3) the “inactive HBV carrier state,” which may follow seroconversion from HBeAg to anti-HBe, is characterized by very low or undetectable serum HBV DNA levels and normal serum aminotransferase levels; (4) “HBeAg-negative CHB” may follow seroconversion from HBeAg to anti-HBe during the immune reactive phase or may develop after years or decades in an inactive carrier state; and (5) the “HBsAg-negative CHB” or “HBsAg-negative” phase.

Although serum levels of alanine transaminase (ALT), an enzyme released by hepatocytes during liver injury, usually reflect the degree of liver damage[3], not all patients with chronic HBV infection have persistently elevated ALT levels. Patients in the immune tolerant phase and inactive HBV carrier state have persistently normal ALT (PNALT) levels, whereas a proportion of patients with HBeAg-negative CHB may have intermittently normal ALT levels. There have also been reports of histological injury in patients with normal ALT levels[4–9]. Furthermore, some large cohort studies have shown that patients with CHB and normal serum ALT levels were at risk of developing cirrhosis and HCC,[10, 11] irrespective of the HBeAg status.

In the present study, we aimed to comprehensively evaluate the characteristics of histological abnormalities in a large population of Chinese patients with CHB and PNALT or persistently or intermittently elevated ALT (PIALT) levels. Moreover, we aimed to analyze the factors associated with significant histological changes in patients with PNALT and PIALT.

## Materials and Methods

### Ethics Statement

The study was approved by the Medical Ethics Committee of The First Affiliated Hospital of Soochow University, and written informed consent was obtained from each patient prior to participation. The study was conducted in accordance with the Declaration of Helsinki.

### Patients

This was a retrospective study of patients with CHB between January 2005 and June 2010 at the Liver Clinic and Department of Hepatosis, The Third Hospital of Zhenjiang Affiliated Jiangsu University. Patients were examined every 3–6 months, or more often if clinically indicated. At each visit, the results of liver biochemistry, ultrasonography, and HBV serology, including HBsAg, HBeAg, anti-HBe, HBV DNA levels, and genotype were assessed. The inclusion criteria were as follows[12]: (1) HBsAg-positive for at least the previous 6 months; (2) HBV DNA level of >1,000 copies/mL; and (3) categorized as having PNALT levels (at least three ALT values taken in the year prior to baseline liver biopsy, of which all values were >40 IU/L and had remained so until the start of treatment or the last follow-up if not treated). Patients were categorized as having PIALT levels if they had at least three ALT values taken, at least one of which was >40 IU/L in the year prior to the baseline biopsy, or any time prior to the start of treatment or last follow-up if not treated (intermittently elevated)[4, 5, 7, 9, 13, 14]. The exclusion criteria were as follows: (1) hepatitis A, C, or D, or human immunodeficiency virus co-infection; (2) evidence of liver disease of another etiology; (3) use of hepatotoxic drugs or regular consumption of alcohol; (4) received previous antiviral (HBV) therapy or any liver functional protection therapy to alleviate hepatic inflammation; and (5) less than three normal ALT values taken prior to the biopsy. When a patient fulfilled the inclusion criteria and had no contraindications to liver biopsy including the presence of ascites, an international normalized ratio of >1.5, activated partial thromboplastin time of >1.5 times, or platelet count of <100,000/mL, the doctor would recommend a liver biopsy. Ultimately, 2303 consecutive patients who underwent liver biopsy were screened. Informed written consent for the liver biopsy was obtained from every patient.

The study protocol was conducted as per the guidelines of the 1975 Declaration of Helsinki and approved by the ethics committee of The Third Hospital of Zhenjiang Affiliated Jiangsu University. Written informed consent was obtained from all subjects.

### Biochemical and Serological Tests

Biochemical tests and complete blood cell counts were performed using routine automated analyzers. The normal upper limit of the ALT level was 40 IU/L. Insulin resistance (IR) was determined using homeostasis model of assessment (HOMA) equations[15], HOMA-IR = [fasting plasma insulin (mIU/l) × fasting plasma glucose (FPG) (mmol/l)]/22.5. HBsAg, HBeAg, and anti-HBe levels were assayed with commercially available enzyme-linked immunosorbent assay (ELISA) kits. The HBV DNA level was measured by real-time PCR, with a lower detection limit of 1000 copies/mL (DaAn Gene Co, shanghai,China).

### Genotype Determination by Multiplex PCR

Genotyping was performed by multiplex PCR using specific primers for each HBV genotype (A–F)[16].

### Liver Biopsy and Histology Assessment

Liver biopsies were obtained using a 16G core aspiration needle with a biopsy length of at least 1.5 cm and included six or more portal tracts. The samples were fixed, paraffin-embedded, and stained with hematoxylin and eosin for morphological evaluation and Masson’s trichrome stain for fibrosis assessment. The pathologist reviewing all biopsy specimens was blinded to the patients’ biochemical and virological results, degree of necrosis and inflammation, and degree of fibrosis according to the Knodell scoring system[17]. Knodell necroinflammatory scores were classified into four categories: minimal (0–3), mild (4–6), moderate (7–9), and severe (10–14) CHB[18]. Minimal and mild scores were considered insignificant, while moderate and severe scores were considered significant. The Knodell fibrosis scores were classified into four categories: minimal (0), mild (1), moderate (2–3), and severe (4). Minimal and mild scores were considered insignificant, while moderate and severe scores were considered significant. Steatosis was graded as follows: 0 (<5% hepatocytes affected); 1 (5–29% of hepatocytes affected); 2 (30–70% of hepatocytes affected); or 3(>70% of hepatocytes affected)[19].

### Statistical Analysis

Results are presented as median (range) or mean ± SD, as appropriate. The patients’ demographic and clinical features were analyzed using the Statistical Package for the Social Sciences (SPSS) version 21.0 (SPSS Inc., Chicago, IL, USA). Statistical analyses were performed using chi-squared and Fisher’s exact tests for categorical variables, while Student’s *t*-test or one-way analysis of variance was used for group comparisons of parametric quantitative data. Multinomial (binary) logistic regression and an ordinal logit model were applied to evaluate factors predicting significant fibrosis based on histology. The predicted probabilities of the parameters were used as a surrogate marker to construct receiver operating characteristic (ROC) curves. ROC and regression analyses were performed using MedCalc (Version 10.4.7.0; MedCalc, Mariakerke, Belgium). All P values were two-sided.

## Results

**Fig 1:**
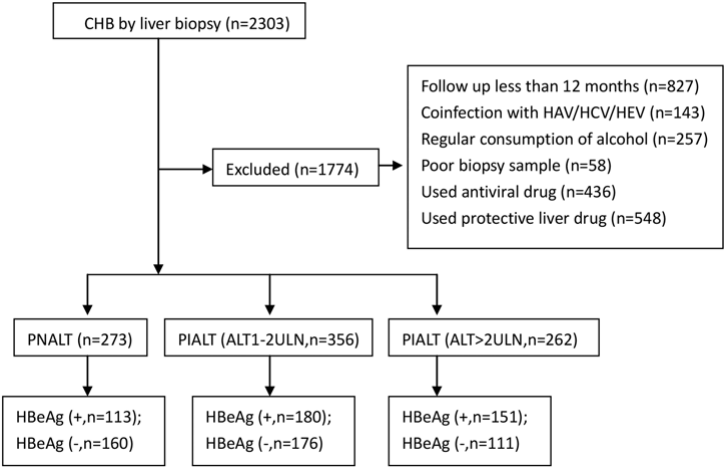
Flow chart of study design.

Among the 2303 patients screened, 891 met the inclusion criteria, including 273 patients with PNALT (113 HBeAg-positive and 160 HBeAg-negative), 356 with PIALT (40< ALT <80 U/L; 180 HBeAg-positive and 176 HBeAg-negative), and 262 with PIALT (ALT >80 U/L; 151 HBeAg-positive and 111 HBeAg-negative). The details of all included and excluded patients are shown in Fig 1.

### Demographic and Clinical Characteristics and Multiple Logistic Regression Analysis Results of HBeAg-Positive Patients

We divided the 444 HBeAg-positive patients into 113 with PNALT, 180 with PIALT (ALT 1–2× upper limit of normal [ULN]), and 151 with PIALT (ALT >2× ULN). The demographic and clinical characteristics of the HBeAg-positive patients are shown in Table 1. Patients with PIALT tended to be older and male and have higher levels of HBV DNA than patients with PNALT. The platelet counts of the patients with PIALT were lower than those with PNALT. Significant necroinflammation was noted in 9.7% (11/113) patients with PNALT, 23.3% (42/180) patients with PIALT (ALT 1–2× ULN), and 27.8% (42/151) patients with PIALT (ALT >2× ULN), whereas significant fibrosis was noted in 8.8% (10/113) patients with PNALT, 27.8% (42/151) patients with PIALT (ALT 1–2× ULN), and 21.2% (32/151) patients with PIALT (ALT >2× ULN). The frequencies of histological abnormalities in patients with PIALT (ALT >2× ULN) or PIALT (ALT 1–2× ULN) were much higher than in those of patients with PNALT (both P<0.05).

**Table 1:** Demographic and clinical characteristics of HBeAg-positive patients and multiple logistic regression analysis of factors associated with ALT.

Patient characteristics	HBeAg-positive						
	PNALT (n = 113)	PIALT (ALT 1–2×ULN, n = 180)	PIALT (ALT>2×ULN, n = 151)	*P* Value	Multivariate*		
					OR	95%CI	*P* Value
Age(years)	32.4 ± 13.2	36.2 ± 11.3	37.5 ± 9.4	**<0.001** ^**a**^	1.08	0.979–1.191	0.126
<30	41 (36.3%)	32 (17.8%)	35 (23.2%)	**0.001** ^**b**^	1		
≥30,<40	56 (49.6%)	83 (46.1%)	68 (45%)		1.813	0.655–5.014	**0.008**
≥40,<60	12 (10.6%)	51 (28.3%)	36 (23.8%)		1.048	0.134–8.177	0.25
≥60	4 (3.5%)	14 (7.8%)	12 (7.9%)		1.124	0.032–8.016	0.357
Sex							
Male	77 (68.1%)	125 (69.4%)	111 (73.5%)	0.59^b^	1		
Female	36 (31.9%)	55 (30.6%)	40 (26.5%)		1.547	0.932–2.565	0.091
BMI	24.12±2.32	25.06±3.11	25.45±3.22	0.353^a^	1.042	0.935–1.162	0.458
<24	82 (72.6%)	115 (63.9%)	98 (64.9%)	0.615^b^	1		
≥24,<28	26 (23%)	54 (30%)	45 (29.8%)		0.973	0.246–1.526	0.643
≥28	5 (4.4%)	11 (6.1%)	8 (5.3%)		1.242	0.243–1.642	0.221
FPG(mmol/L)	5.12±1.46	5.21±1.62	5.26±1.55	0.728	0.924	0.824–1.035	0.792
HOMA-IR(mU/L)	10.13±2.37	11.53±3.25	11.63±2.67	0.326	0.482	0.537–1.482	0.248
PLT (×10^9^/L)	207.3 ± 64.8	201.8 ± 55.4	196.3 ± 63.1	0.118^a^	0.997	0.988–1.007	0.604
PTA (%)	99.4 ± 6.3	98.6 ± 8.7	101.3 ± 11.7	0.277 ^a^	1.024	0.944–1.111	0.57
ALB (g/L)	42.3 ± 3.5	41.4 ± 4.7	43.3 ± 4.5	0.513 ^a^	0.995	0.913–1.084	0.902
ALT (U/L)	27.4 ± 6.5	53.2 ± 18.7	102.6 ± 25.4	**<0.001** ^**a**^			
AST (U/L)	23.5 ± 7.4	45.4 ± 12.9	74.5 ± 22.6	**<0.001** ^**a**^	1.908	1.382–2.633	**<0.001**
HBsAg (LgIU/L)	4.41 ± 0.73	4.32± 0.68	4.28 ± 0.56	**<0.001** ^**a**^	1.136	0.251–2.142	**0.003**
HBV DNA (Lgcopies/mL)	7.42±2.43	6.73±2.533	6.64±2.46	**0.047** ^**a**^	1.733	0.758–3.964	0.193
≥3,<5	12 (10.6%)	21(11.7%)	32 (21.2%)	**0.001** ^**b**^	1		
≥5,<7	45 (39.8%)	101 (56.1%)	76 (50.3%)		1.18	0.066–2.113	0.556
≥7	56(49.6%)	58(32.2%)	43(28.5%)		2.132	0.327–5.373	**0.015**
Genotype							
B	21 (18.6%)	34 (18.9%)	24 (15.9%)	0.753^b^	1		
C	92 (81.4%)	146 (81.1%)	127 (84.1%)		0.678	0.364–1.261	0.22
Necroinflammatory scores	5.56±2.64	6.37±3.62	7.54±3.54	**0.001** ^**a**^	2.143	1.112–4.264	**0.023**
minimal	47 (41.6%)	24 (13.3%)	22 (14.6%)	**<0.001** ^**c**^	1		
mild	55 (48.7%)	113 (62.8%)	87 (57.6%)				
moderate	11 (9.7%)	35 (19.4%)	31 (20.5%)		2.542	1.485–8.513	**<0.001**
severe	0	8 (4.4%)	11 (7.3%)				
fibrosis scores	1.67±0.43	2.27±1.22	2.12±1.25	**0.007** ^**a**^	1.536	0.874–2.154	**0.001**
minimal	36 (31.9%)	21 (11.7%)	24 (15.9%)	**<0.001** ^**b**^	1		
mild	67 (59.3%)	117 (65%)	95 (62.9%)				
moderate	9 (8.0%)	37 (20.6%)	26 (17.2%)		1.464	0.154–3.164	**<0.001**
severe	1 (0.9%)	5 (2.8%)	6 (4.0%)				
steatosis							
0	68 (60.2%)	113 (62.8%)	95 (62.9%)	0.634^b^			
1	37 (32.7%)	53 (29.4%)	48 (25.2%)		0.765	0.964–1.026	0.773
2	7 (6.2%)	11 (6.1%)	16 (10.6%)		1.036	0.947–1.113	0.593
3	1 (0.9%)	3 (1.7%)	2 (1.3%)		1.135	0.648–1.374	0.538

Parameters are expressed as mean±SD or number (%).

PNALT, persistent normal ALT; PLT, platelet; PTA, prothrombin activity; ALB, albumin; ALT, alanine aminotransaferase; AST, aspirate aminotransferase; ULN, upper limit of the normal range; BMI, body mass index, China BMI categories are: (i) <18.5 kgm -2: underweight; (ii) 18.5~24 kgm -2: normal weight; (iii) 24~28 kgm -2: pre-obese;and (iv) 28 kgm -2: obesity. FPG, fasting plasma glucose; HOMA-IR, homeostasis model assessment of insulin resistance.

The normal range of ALT and AST are 5–40 U/L, PLT is 100–300 ×109/L, ALB is 35–55 g/L; The Knodell necroinflammatory scores were classified into 4 categories: minimal (0–3), mild (4–6), moderate (7–9), and severe (10–14) chronic hepatitis, The Knodell fibrosis scores were classified into 4 categories: minimal (0), mild (1), moderate (2–3), and severe (4) fibrosis.

^a^One-way analysis.

^b^Pearson Chi-Square.

^c^Fisher exact tests.

*Binary logistic regression, enter method.

We found that age, aspartate aminotransferase (AST) level, HBsAg level, HBV DNA level, and histological abnormalities differed significantly between the PNALT and PIALT groups. When PNALT/PIALT was considered as a binary dependent variable, we used multiple logistic (binary) regression analysis to assess the factors associated with PNALT and PIALT in HBeAg-positive patients (Table 1). The results suggested that age of 30–40 years, HBsAg level, HBV DNA≥10^7^copies/Land AST level were associated with PNALT and PIALT in HBeAg-positive patients. We also found significant histological abnormalities associated with PNALT and PIALT.

### Demographic and Clinical Characteristics and Multiple Logistic Regression Analysis Results of HBeAg-Negative Patients

We divided the 447 HBeAg-negative patients into 160 with PNALT, 176 with PIALT (ALT 1–2× ULN), and 111 with PIALT (ALT >2× ULN). The demographic and clinical characteristics of HBeAg-positive patients are shown in Table 1. Patients with PIALT levels tended to be older and male and have a higher body mass index (BMI) than patients with PNALT. Moreover, the BMI of patients with PIALT (ALT >2× ULN) or PIALT (ALT 1–2× ULN) was much higher than in patients with PNALT (both P<0.05), while the platelet counts of patients with PIALT were lower than those of patients with PNALT. Significant necroinflammation was observed in 10% (16/113) patients with PNALT, 23.9% (42/176) patients with PIALT (ALT 1–2× ULN), and 27.9% (31/111) patients with PIALT (ALT >2× ULN), whereas significant fibrosis was noted in 14.4% (23/160) patients with PNALT, 26.7% (47/176) patients with PIALT (ALT 1–2× ULN), and 28.8% (32/111) patients with PIALT (ALT >2× ULN). The frequencies of histological abnormalities in patients with PIALT (ALT >2× ULN) or PIALT (ALT 1–2× ULN) were much higher than those of patients with PNALT (both P<0.05).

We found that age, AST level, HBsAg level, and histological abnormalities differed significantly between the PNALT and PIALT groups. When PNALT/PIALT was considered as a binary dependent variable, we used multiple logistic (binary) regression analysis to assess factors associated with PNALT and PIALT in HBeAg-negative patients (Table 2). Using the “enter” method, the results suggested that an age of 40–60 years, AST level, and albumin level were associated with significant histological abnormalities in patients with PNALT and PIALT.

**Table 2:** Demographic and clinical characteristics of HBeAg-negative patients and multiple logistic regression analysis of factors associated with ALT.

Patient characteristics	HBeAg-negative						
	PNALT (n = 160)	PIALT (ALT 1–2×ULN, n = 176)	PIALT (ALT >2×ULN, n = 111)	*P* Value	Multivariate*		
					OR	95%CI	*P* Value
Age(years)	36.4 ± 10.5	36.5 ± 11.6	38.5 ± 13.2	**<0.001** ^**a**^	1.436	0.635–2.146	**0.001**
<30	52 (32.5%)	32 (18.2%)	33 (29.2%)	**0.049** ^**b**^		1	
≥30,<40	56 (35%)	72 (40.9%)	44 (40.7%)		1.006	0.122–8.285	0.906
≥40,<60	45 (28.1%)	56 (31.8%)	26 (23.0%)		4.588	1.400–15.036	**0.012**
≥60	7 (4.4%)	16 (9.1%)	8 (3.1%)		1.65	0.025–10.969	0.815
Sex							
Male	108 (67.5%)	128 (72.7%)	80 (72.1%)	0.538^b^		1	
Female	52 (32.5%)	48 (27.3%)	31 (27.9%)		0.936	0.464–1.899	0.855
BMI	23.25±2.27	24.67±3.01	25.21±2.26	0.025^a^	1.035	0.893–1.199	0.646
<24	116 (72.5%)	100 (56.8%)	69 (62.2%)	**0.034** ^**b**^		1	
≥24,<28	38 (23.8%)	60 (34.1%)	32 (28.8%)		1.025	0.453–1.004	0.483
≥28	6 (3.8%)	16 (9.1%)	10 (9%)		1.004	0.073–1.180	0.287
FPG(mmol/L)	5.30±2.64	5.42±3.42	5.22±2.11	0.427	0.638	0.554–1.254	0.432
HOMA-IR(mU/L)	9.84±3.64	11.35±4.27	11.53±3.74	0.311	0.482	0.663–1.537	0.226
PLT (×10^9^/L)	196.3 ± 76.1	188.4± 66.7	182.4±70.3	**0.025** ^**a**^	0.987	0.974–1.000	0.053
PTA (%)	103.2 ± 8.9	98.6 ± 11.4	101.5± 8.5	0.132^a^	1.065	0.950–1.194	0.281
ALB (g/L)	42.5 ± 4.4	41.4 ± 4.4	42.3 ± 4.7	0.473^a^	1.209	1.058–1.380	**0.005**
ALT (U/L)	23.8 ± 8.0	54.6 ± 13.5	94.5 ± 18.4	**<0.001** ^**a**^			
AST (U/L)	23.9±5.7	78.9±58.0	84.5 ± 31.6	**<0.001** ^**a**^	1.139	1.107–1.173	**<0.001**
HBsAg (LgIU/L)	3.51± 0.43	3.75 ± 0.23	4.04 ± 0.45	**0.025** ^**a**^	2.135	1.326–2.537	**<0.001**
HBV DNA (Lgcopies/mL)	5.02±1.28	5.63±1.21	6.01±1.82	0.066^a^	0.919	0.704–1.201	0.538
≥3,<5	45 (28.1%)	51(29%)	22 (19.8%)	0.19^b^		1	
≥5	115 (71.9%)	125 (71%)	89 (80.2%)		1.243	0.932–1.352	0.364
Genotype							
B	45 (28.1%)	64 (36.4%)	26 (23.4%)	0.052^b^		1	
C	115 (71.9%)	112 (63.6%)	85 (76.6%)		1.377	0.557–3.305	0.489
Necroinflammatory scores	5.26±2.53	6.14±2.26	7.18±2.53	**0.027** ^**a**^	1.132	0.854–3.153	**0.012**
minimal	62 (38.8%)	37 (21%)	12 (10.8%)	**<0.001** ^**b**^		1	
mild	81 (50.6%)	95 (54%)	68 (61.3%)				
moderate	15 (9.4%)	37 (21%)	22 (19.8%)		1.583	1.002–3.523	**0.013**
severe	1 (0.6%)	7 (4%)	9 (8.1%)				
fibrosis scores	1.52±0.53	2.33±1.03	2.54±1.65	**<0.001** ^**a**^	3.171	1.051–9.604	**0.029**
minimal	57 (35.6%)	28 (15.9%)	24 (21.6%)	**<0.001** ^**b**^		1	
mild	80 (50%)	101 (57.4%)	55 (49.5%)				
moderate	20 (12.5%)	35 (19.9%)	21 (18.9%)		3.269	1.366–7.281	**0.008**
severe	3 (1.9%)	12 (6.8%)	11 (9.9%)				
steatosis							
0	103 (64.4%)	110 (62.5%)	76(68.5%)	0.86		1	
1	47 (29.4%)	50 (28.4%)	27 (24.3%)		0.934	0.792–1.116	0.464
2	9 (5.6%)	13 (7.4%)	6 (5.4%)		1.002	0.976–1.043	0.794
3	1 (0.6%)	3 (1.7%)	2 (1.8%)		1.026	0.869–1.226	0.583

^a^Independent Samples T Test.

^b^Pearson Chi-Square.

*Binary logistic regression, enter method.

### Analysis of Factors Associated with Significant Necroinflammation in HBeAg-Positive and -Negative Patients with PNALT

When the presence or absence of significant necroinflammation was considered as a binary dependent variable in HBeAg-positive and -negative patients with PNALT, univariate analysis indicated that ALT and age were associated with significant histological abnormalities in HBeAg-positive and -negative patients with PNALT, respectively (Tables 3 and 4). However, multiple logistic regression analysis indicated that no parameter was associated with significant histological abnormalities in patients with PNALT, regardless of the HBeAg-positive or -negative status (Tables 3 and 4).

**Table 3:** Multiple logistic regression analysis of factors associated with significant necroinflammatory in HBeAg-positive PNALT patients.

Parameter	No significance (n = 102)	Significance (n = 11)	*P*	Multivariate*			
				OR	95%CI	Wald	*P*
Age(years)	32.15±8.33	33.04±9.66	0.132^a^	0.828	0.533–1.288	0.008	0.403
<30	39(38.2%)	2(18.2%)	0.336^b^		1		
≥30,<40	52(51.0%)	6(54.5%)		4.631	0.488–43.905	1.784	0.182
≥40,<60	8(7.8%)	2(18.2%)		16.241	0.0570–1038.538	1.897	0.168
≥60	3(2.9%)	1(9.1%)		14.286	0.028–683.815	1.229	0.268
Sex							
Female	32(31.7%)	4(36.4%)	0.736^b^		1		
Male	70(68.3%)	7(63.6%)		0.414	0.073–2.244	0.995	0.319
BMI	24.13±2.22	24.46±2.22	0.143^a^	0.757	0.503–1.14	1.775	0.183
PLT (×10^9^/L)	205.36±24.36	201.56±28.45	0.346^a^	0.999	0.987–1.045	1.619	0.203
PTA (%)	99.66±2.47	100.64±2.351	0.887^a^	1.215	0.872–1.512	0.969	0.325
ALB (g/L)	43.26±3.64	43.75±4.35	0.463^a^	0.908	0.772–1.538	1.012	0.839
ALT (U/L)	25.97±7.68	27.43±7.88	0.154^a^	1.026	0.791–1.221	0.025	0.588
≤0.75× ULN	71(69.6%)	4(36.4%)	**0.027** ^**b**^		1		
0.75–1 × ULN	31(30.4%)	7(63.6%)		9.852	0.509–190.44	2.29	0.13
AST (U/L)	19.27±6.33	20.15±6.43	0.496^a^	1.017	0.840–1.013	0.968	0.573
HBsAg (LgIU/L)	4.20 ± 0.63	4.26±0.47	0.648	0.487	0.574–1.423	1.043	0.864
HBV DNA (Lgcopies/mL)	7.11±2.15	6.85±2.64	0.58^a^	0.628	0.201–1.967	0.642	0.423
≥3,<5	10(9.8%)	2(18.2%)	0.662^b^		1		
≥5,<7	36(35.3%)	4(36.4%)		1.651	0.353–7.712	0.345	0.524
≥7	56(54.9%)	5(45.5%)		1.024	0.883–1.027	0.634	0.772
Genotype							
B	16(15.7%)	4(36.4%)	0.088^b^		1		
C	86(84.3%)	7(63.6%)		3.536	0.325–11.642	1.171	0.279

^a^Independent Samples T Test.

^b^Pearson Chi-Square.

*Binary logistic regression, enter method.

**Table 4:** Multiple logistic regression analysis of factors associated with significant necroinflammatory in HBeAg-negative PNALT patients.

Parameter	No significance (n = 144)	Significance (n = 16)	*P*	Multivariate*			
				OR	95%CI	Wald	*P*
Age(years)	36.22±8.33	38.13±10.43	**0.001** ^**a**^	1.242	0.782–1.321	0.014	0.363
<30	50(34.7%)	2(12.5%)	**0.015** ^**b**^		1		
≥30,<40	52(36.1%)	5(31.3%)		2.142	0.242–5.125	1.362	0.554
≥40,<60	38(26.4%)	6(37.5%)		4.275	0.024–18.390	1.544	0.223
≥60	4(2.8%)	3(18.8%)		11.223	0.132–120.432	0.362	0.448
Sex							
Female	46(31.9%)	6(37.5%)	0.653^b^		1		
Male	98(68.1%)	10(62.5%)		0.553	0.643–1.214	1.122	0.445
BMI	24.03±2.17	24.83±3.11	0.536	1.034	0.642–1.453	0.111	0.835
PLT (×10^9^/L)	197.35±23.97	189.56±27.78	0.332^a^	0.879	0.924–1.042	4.753	0.856
PTA (%)	99.98±2.83	100.46±2.701	0.403^a^	1.006	0.875–1.154	0.132	0.332
ALB (g/L)	42.97±3.65	41.5±4.68	0.776^a^	0.965	0.742–1.214	1.227	0.335
ALT (U/L)	23.24±7.25	25.56±8.45	0.329^a^	1.112	0.563–1.476	0.031	0.556
≤0.75× ULN	75(52.1%)	7(43.8%)	0.527^b^		1		
0.75–1 × ULN	69(47.9%)	9(56.2%)		0.643	0.215–2.146	0.436	0.352
AST (U/L)	23.537±8.34	24.34±6.77	0.665^a^	1.017	0.760–1.215	0.832	0.886
HBsAg (LgIU/L)	3.44±0.56	3.56±0.57	0.216	0.846	0.784–1.326	1.536	0.643
HBV DNA (Lgcopies/mL)	4.97±1.54	5.01±1.21	0.58^a^	1.42	0.793–2.544	0.232	0.238
≥3,<5	37(25.7%)	7(43.8%)	0.125^b^		1		
≥5	107(74.3%)	9(56.2%)		1.651	0.442–6.453	0.453	0.675
Genotype							
B	39(27.1%)	6(37.5%)	0.379^b^		1		
C	105(72.9%)	10(62.5%)		4.143	0.170–21.342	1.023	0.302

^a^Independent Samples T Test.

^b^Pearson Chi-Square.

*Binary logistic regression, enter method.

### Analysis of Factors Associated with Significant Fibrosis in HBeAg-Positive and -Negative Patients with PNALT

When the presence or absence of significant fibrosis was considered as a binary dependent variable in HBeAg-positive and -negative patients with PNALT, univariate and multiple logistic regression analyses indicated that no parameter was associated with significant fibrosis in HBeAg-positive patients with PNALT (Table 5). However, in HBeAg-negative patients with PNALT, univariate analysis indicated that age and PLT level were associated with significant fibrosis, whereas multiple logistic regression analysis indicated that age 40–60 years was associated with significant fibrosis (Table 6).

**Table 5:** Multiple logistic regression analysis of factors associated with significant fibrosis in HBeAg-positive PNALT patients.

Parameter	No significance (n = 103)	Significance (n = 10)	*P*	Multivariate*			
				OR	95%CI	Wald	*P*
Age(years)	32.25±6.75	32.66±9.34	0.574^a^	0.642	0.426–1.164	0.012	0.643
<30	36(35%)	2(20%)	0.375^b^		1		
≥30,<40	55(53.4%)	5(50%)		3.542	0.521–13.254	1.534	0.435
≥40,<60	9(8.7%)	2(20%)		11.226	0.065–137.53	1.322	0.354
≥60	3(2.9%)	1(10%)		7.233	0.054–66.63	1.534	0.343
Sex							
Female	33(32%)	3(30%)	0.895^b^		1		
Male	70(68%)	7(70%)		0.532	0.147–1.647	0.853	0.547
BMI	24.21±2.13	24.31±2.13	0.146^a^	0.436	0.124–1.006	1.075	0.443
PLT (×10^9^/L)	204.37±24.36	198.64±31.35	0.643^a^	0.876	0.8357–1.354	1.446	0.374
PTA (%)	99.46±2.54	99.86±2.31	0.904^a^	1.231	0.843–1.556	0.667	0.432
ALB (g/L)	43.24±3.73	42.16±4.55	0.557^a^	0.889	0.674–1.327	0.896	0.547
ALT (U/L)	25.22±8.45	26.34±8.55	0.253^a^	1.216	0.536–1.625	0.034	0.646
≤0.75× ULN	64(62.1%)	4(40%)	0.894^b^		1		
0.75–1 × ULN	39(37.9%)	6(60%)		4.365	0.643–22.534	1.543	0.113
AST (U/L)	19.46±6.21	19.89±6.43	0.889^a^	1.002	0.864–1.007	0.984	0.657
HBsAg (LgIU/L)	4.16 ± 0.56	4.22±0.57	0.574	0.112	0.475–1.153	0.886	0.474
HBV DNA (Lgcopies/mL)	7.05±2.64	6.89±2.43	0.644^a^	0.576	0.645–1.2147	0.543	0.674
≥3,<5	10(9.7%)	2(20%)	0.336^b^		1		
≥5,<7	38(36.9%)	2(20%)		1.578	0.437–6.354	0.332	0.566
≥7	55 (53.4%)	6 (60%)		1.027	0.859–1.073	0.538	0.853
Genotype							
B	33(32%)	4(36.4%)	0.609^b^		1		
C	70(68%)	6(63.6%)		1.146	0.545–1.654	0.543	0.653

^a^Independent Samples T Test.

^b^Pearson Chi-Square.

*Binary logistic regression, enter method.

**Table 6:** Multiple logistic regression analysis of factors associated with significant fibrosis in HBeAg-negative PNALT patients.

Parameter	No significance (n = 137)	Significance (n = 23)	*P*	Multivariate*			
				OR	95%CI	Wald	*P*
Age(years)	35.52±7.56	40.34±11.45	**<0.001** ^**a**^	2.143	0.553–6.453	1.543	**0.032**
<30	50(38.5%)	2(8.7%)	**0.003** ^**b**^		1		
≥30,<40	49(34.5%)	7(30.4%)		2.443	0.242–7.345	1.423	0.065
≥40,<60	34(23.9%)	11(47.8%)		3.223	0.124–14.344	2.153	**0.005**
≥60	4(2.8%)	3(13%)		14.223	0.112–246.75	0.653	0.477
Sex							
Female	43(31.4%)	9(34.8%)	0.463^b^		1		
Male	94(68.6%)	14(65.2%)		0.526	0.537–1.843	1.034	0.536
BMI	24.36±2.43	24.45±2.45	0.352	1.186	0.758–1.235	0.086	0.887
PLT (×10^9^/L)	198.45±24.45	182.56±28.45	**0.034** ^**a**^	0.879	0.647–2.654	2.753	0.152
PTA (%)	99.43±2.13	99.86.46±2.45	0.985^a^	1.004	0.875–1.044	0.057	0.785
ALB (g/L)	43.05±3.23	42.53±2.53	0.463^a^	0.647	0.465–1.632	1.135	0.432
ALT (U/L)	24.64±5.74	25.75±8.43	0.354^a^	1.242	0.574–1.735	0.536	0.438
≤0.75× ULN	68(49.6%)	14(60.9%)	0.319^b^		1		
0.75–1 × ULN	69(50.4%)	9(39.1%)		0.665	0.426–1.645	0.476	0.537
AST (U/L)	23.64±8.34	24.646.36	0.646^a^	1.314	0.722–1.463	0.647	0.843
HBsAg (LgIU/L)	3.43±0.66	3.53±0.63	0.266	0.864	0.707–1.364	1.557	0.675
HBV DNA (Lgcopies/mL)	4.75±1.65	4.95±1.26	0.647^a^	1.643	0.463–1.755	0.634	0.247
≥3,<5	34(24.8%)	10(38.5%)	0.151^b^		1		
≥5	103(75.2%)	16(61.5%)		1.425	0.428–1.254	0.546	0.664
Genotype							
B	39(27.1%)	9(39.1%)	0.236^b^		1		
C	105(72.9%)	14(60.9%)		2.123	0.132–5.563	0.843	0.246

^a^Independent Samples T Test.

^b^Pearson Chi-Square.

*Binary logistic regression, enter method.

### Area Under the Curve (AUC) of ALT Level Associated with Significant Histological Abnormalities in HBeAg-Positive and -Negative Patients

**Fig 2:**
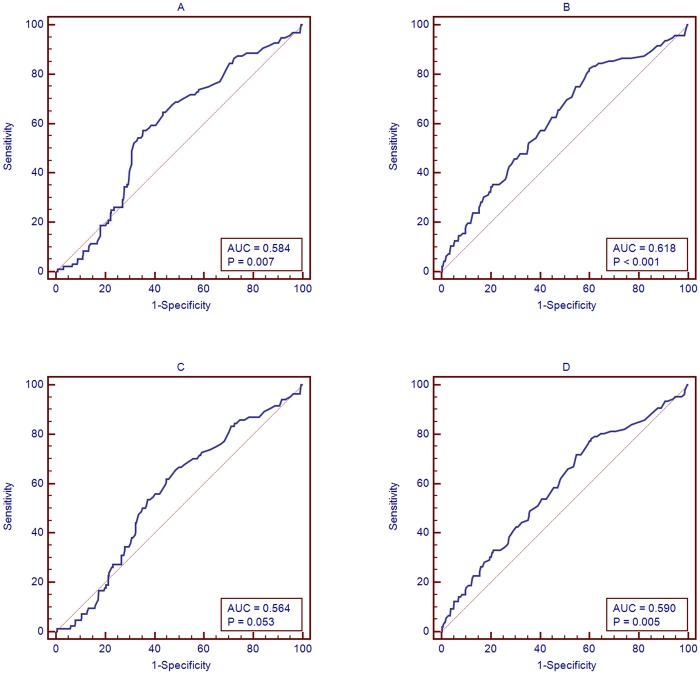
AUC of ALT associated with significant histological characteristics in HBeAg positive and HBeAg negative patients. A: AUC of ALT associated with significant necroinflammatory scores in HBeAg positive patients; B: AUC of ALT associated with significant fibrosis scores in HBeAg positive patients; C: AUC of ALT associated with significant necroinflammatory scores in HBeAg negative patients; D: AUC of ALT associated with significant fibrosis scores in HBeAg negative patients.

ALT levels were used to predict the probability of being diagnosed with significant histological abnormalities in HBeAg-positive and -negative patients. The AUC of the ALT level associated with the diagnosis of significant necroinflammation in HBeAg-positive patients was 0.584 (95% confidence interval [CI], 0.523–0.645; sensitivity = 57.3%, specificity = 64.7%, P = 0.007; Fig 2A). The AUC of the ALT level associated with the diagnosis of significant necroinflammation in HBeAg-negative patients was 0.618 (95% CI, 0.554–0.681; sensitivity = 82.3%, specificity = 39.9%, P<0.001; Fig 2B). The AUC of the ALT level associated with the diagnosis of significant fibrosis in HBeAg-positive patients was 0.564 (95% CI, 0.523–0.645; sensitivity = 61.9%, specificity = 55.1%, P = 0.0526; Fig 2C). The AUC of the ALT level associated with the diagnosis of significant fibrosis in HBeAg-negative patients was 0.59 (95% CI, 0.527–0.653; sensitivity = 79.3%, specificity = 39.3%, P = 0.0049; Fig 2D).

### AUC of Age Associated with Significant Histological Fibrosis in HBeAg-Positive and -Negative Patients with PNALT

**Fig 3:**
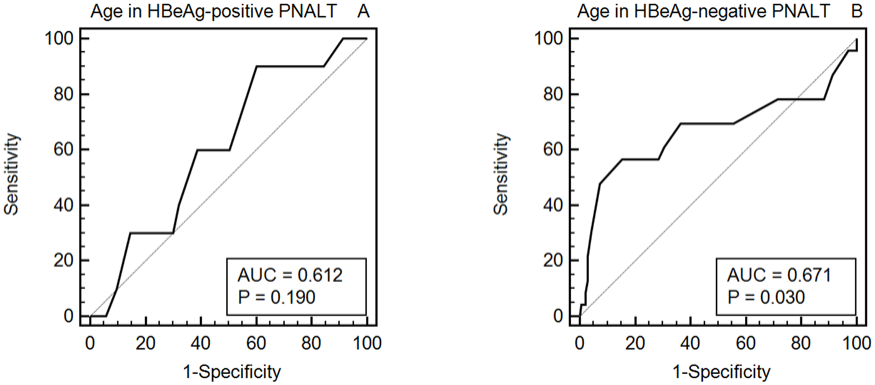
AUC of age associated with significant fibrosis in HBeAg-positive and HBeAg-negative PNALT patients. A: AUC of age associated with significant fibrosis in HBeAg-positive PNALT patients. B: AUC of age associated with significant fibrosis in HBeAg-negative PNALT patients.

Age was used to predict the probability of being diagnosed with significant fibrosis abnormalities in HBeAg-positive and -negative patients with PNALT. The AUC of the age associated with the diagnosis of significant fibrosis abnormalities in HBeAg-positive PNALT patients was 0.612 (95% CI, 0.516–0.702; sensitivity = 54.5%, specificity = 64.6%, P = 0.192; Fig 3A). The AUC of the age associated with the diagnosis of significant fibrosis in HBeAg-negative patients with PNALT was 0.672 (95% CI, 0.593–0.74; sensitivity = 66.7%, specificity = 75.4%, P = 0.032; Fig 3B).

## Discussion

Despite its invasive nature, liver biopsy remains the gold standard for assessing histological liver abnormalities[20]. The 2012 Asian-Pacific Association for the Study of the Liver (APASL) guidelines recommended that liver biopsies should be considered in patients with viremic CHB aged >40 years, especially in those with high normal or minimally increased ALT levels[1, 21]. Previous studies focusing on the histological characteristics of the liver in such cases have been limited by a small sample size, a focus on patients with elevated ALT levels, or a follow-up time of <6–12 months[22–25]. Therefore, in the present study, we considered HBV DNA levels of >1000 copies/mL and limited ALT level assessments to at least every 3 months for at least 1 year[26]. We aimed to comprehensively evaluate the histological characteristics of a large population of Chinese patients with CHB and PNALT or elevated ALT levels.

Some reports indicated that HBeAg-positive patients with PNALT usually have high levels of HBV DNA with no or minimal liver histological changes[27, 28]. We examined the histological features in HBeAg-positive patients with PNALT and PIALT. Although these patients had lower active significant necroinflammatory and fibrosis scores than HBeAg-positive patients with elevated ALT levels, our results demonstrate that significant necroinflammatory and fibrosis scores were found in HBeAg-positive patients with PNALT (9.7% and 8%). We also found similar histological features in HBeAg-negative patients with PNALT and PIALT. The frequencies of the histological abnormalities in patients with PIALT (ALT >2× ULN) or PIALT (ALT 1–2× ULN) were much higher than in patients with PNALT (both P<0.05). Andreani *et al*. retrospectively demonstrated mild (F1) fibrosis in 20 of 40 patients during the follow-up period. During a median follow-up period of 37.7 months, only 9.7% (3/31) of patients developed chronic hepatitis[27]. Seto *et al*. found that 22.5% of patients had significant histological abnormalities[22]. Furthermore, Lesmana *et al*. showed that 40.2% (in India) and 68.6% (in Indonesia) of these patients had significant fibrosis[7, 29]. These studies reported more significant histological abnormalities than that observed in the present study. We believe that the reason for this is that these patients had elevated ALT levels at follow-up, whereas the histological abnormality results were similar to those of our patients with PIALT. A systematic review[5] illustrated 215 patients with serum HBV DNA levels of <20,000 IU/mL from four studies with good or acceptable definitions of PNALT[7, 30–32]. Necroinflammatory activity was more than just minimal in five (7%) and fibrosis was more than just mild in seven (10%) of 73 patients with serum HBV DNA levels of 2000–20,000 IU/mL as well as in two (1.4%) and one (0.7%) of 142 patients with serum HBV DNA levels of <2000 IU/mL, respectively.

However, the 2012 EASL guidelines recommended that liver biopsies should be considered in immune tolerant patients aged >30 years in addition to patients with fluctuating ALT levels[26]. Advice on which patients should be recommended for liver biopsy, especially those with normal ALT levels, vary among these guidelines. Multiple logistic regression analysis of age revealed that an age of 30–40 years was associated with PNALT and PIALT in patients with CHB if the AST level was excluded. We stratified patients based on an age of 30 years according to the EASL guidelines. We found significant necroinflammation in 4.3% (4/93) and significant fibrosis in 4.4% (4/90) patients with PNALT. The results suggest that patients aged >30 years in the PIALT group had a higher frequency of necroinflammation (25.89%, 160/618) than those aged <30 years in the PNALT group (9.89%, 27/273; P<0.001). Similarly, patients aged >30 years in the PIALT group had a higher frequency of necroinflammation (23.75%, 153/618) than those aged <30 years in the PNALT group (9.89%, 33/273, P<0.001).

One study by Wong *et al*. from Hong Kong indicated that the risk of liver fibrosis increases after the age of 35 years in HBeAg-positive patients[33]. Nevertheless, patients with advanced fibrosis (F3–F4) were identified based on transient elastography measurement instead of histological evidence. Interestingly, we found that the AUC of the age associated with the diagnosis of significant fibrosis in HBeAg-negative patients with PNALT had a cutoff value of 42 years.

Zoulim *et al*. recently suggested starting therapy in all patients with normal ALT levels who show relatively low levels of viremia, not just in patients aged >40 years, but also among patients in their 20s. This represents a step further than that reported in the major guidelines. The author demonstrated that many unnecessary deaths might be prevented by earlier antiviral intervention; additionally, considering its long duration, initiation and promotion may both be significant during the immune tolerant phase and may increase the risk of HCC later in life, even in the absence of cirrhosis[34]. However, in a prospective randomized, double-blind, placebo-controlled study, 380 patients with CHB and PNALT were screened while receiving entecavir therapy for 1 year and achieved virological improvements but no histological benefits[35]. Therefore, we supported the proposal that patients with CHB and PNALT usually have high levels of HBV DNA with no or minimal liver histological changes. In addition, trials have shown that these patients tend to not have a good response to the currently available antiviral therapy[36, 37].

ALT, as a biomarker of liver injury, also predicts sensitivity and specificity in liver disease and is associated with significant histological characteristics to some extent in HBeAg-positive and -negative patients. The AUC of the ALT level associated with the diagnosis of significant necroinflammation in HBeAg-positive patients was 0.584 (P = 0.007). The AUC of the ALT level associated with diagnosis of significant necroinflammation in HBeAg-negative patients was 0.618 (P = 0.003). The AUC of the ALT level associated with the diagnosis of significant fibrosis in HBeAg-negative patients was 0.59 (P = 0.0049). Obviously, from these results, it can be concluded that the ALT level was also good at predicting the probability of a diagnosis of significant histological characteristics in PIALT patients compared to PNALT patients.

In conclusion, significant histological abnormalities are not often observed in Chinese patients with PNALT levels when good or acceptable definitions of PNALT are used. However, ALT level is a predictor of significant histological characteristics between PNALT and elevated ALT levels. Interestingly, age appears to be a predictor of a diagnosis of significant fibrosis in HBeAg-negative patients with PNALT.

## References

[1] Yan H, Zhong G, Xu G, He W, Jing Z, Gao Z, et al Sodium taurocholate cotransporting polypeptide is a functional receptor for human hepatitis B and D virus. eLife. 2012;1:e00049 10.7554/eLife.00049 23150796 PMC3485615

[2] McMahon BJ. The natural history of chronic hepatitis B virus infection. Semin Liver Dis. 2004;24 Suppl 1:17–21. Epub 2004/06/12. 10.1055/s-2004-828674 . 15192797

[3] Kim WR, Flamm SL, Di Bisceglie AM, Bodenheimer HC. Serum activity of alanine aminotransferase (ALT) as an indicator of health and disease. Hepatology. 2008;47(4):1363–70. Epub 2008/03/28. 10.1002/hep.22109 . 18366115

[4] Wang H, Xue L, Yan R, Zhou Y, Wang MS, Cheng MJ, et al Comparison of histologic characteristics of Chinese chronic hepatitis B patients with persistently normal or mildly elevated ALT. PloS one. 2013;8(11):e80585 10.1371/journal.pone.0080585 24260428 PMC3832452

[5] Papatheodoridis GV, Manolakopoulos S, Liaw YF, Lok A. Follow-up and indications for liver biopsy in HBeAg-negative chronic hepatitis B virus infection with persistently normal ALT: a systematic review. J Hepatol. 2012;57(1):196–202. Epub 2012/03/28. 10.1016/j.jhep.2011.11.030 S0168–8278(12)00234–6 [pii]. . 22450396

[6] Lai M, Hyatt BJ, Nasser I, Curry M, Afdhal NH. The clinical significance of persistently normal ALT in chronic hepatitis B infection. J Hepatol. 2007;47(6):760–7. Epub 2007/10/12. S0168–8278(07)00476-X [pii] 10.1016/j.jhep.2007.07.022 . 17928090

[7] Kumar M, Sarin SK, Hissar S, Pande C, Sakhuja P, Sharma BC, et al Virologic and histologic features of chronic hepatitis B virus-infected asymptomatic patients with persistently normal ALT. Gastroenterology. 2008;134(5):1376–84. Epub 2008/05/13. 10.1053/j.gastro.2008.02.075 S0016–5085(08)00356–9 [pii]. . 18471514

[8] Lin CL, Liao LY, Liu CJ, Yu MW, Chen PJ, Lai MY, et al Hepatitis B viral factors in HBeAg-negative carriers with persistently normal serum alanine aminotransferase levels. Hepatology. 2007;45(5):1193–8. Epub 2007/04/28. 10.1002/hep.21585 . 17464993

[9] Dai CY, Chuang WL, Huang JF, Yu ML. Hepatitis B e antigen-negative patients with persistently normal alanine aminotransferase levels and hepatitis B virus DNA >2000 IU/mL. Hepatology. 2009;49(2):704–5; author reply 5–6. Epub 2009/01/30. 10.1002/hep.22723 . 19177587

[10] Yuen MF, Yuan HJ, Wong DK, Yuen JC, Wong WM, Chan AO, et al Prognostic determinants for chronic hepatitis B in Asians: therapeutic implications. Gut. 2005;54(11):1610–4. Epub 2005/05/06. gut.2005.065136 [pii] 10.1136/gut.2005.065136 15871997 PMC1774768

[11] Chen CJ, Yang HI, Su J, Jen CL, You SL, Lu SN, et al Risk of hepatocellular carcinoma across a biological gradient of serum hepatitis B virus DNA level. JAMA. 2006;295(1):65–73. Epub 2006/01/05. 295/1/65 [pii] 10.1001/jama.295.1.65 . 16391218

[12] Liao B, Wang Z, Lin S, Xu Y, Yi J, Xu M, et al Significant fibrosis is not rare in Chinese chronic hepatitis B patients with persistent normal ALT. PloS one. 2013;8(10):e78672 10.1371/journal.pone.0078672 24205292 PMC3808379

[13] Wang H, Xue L, Yan R, Zhou Y, Wang MS, Cheng MJ, et al Comparison of FIB-4 and APRI in Chinese HBV-infected patients with persistently normal ALT and mildly elevated ALT. J Viral Hepat. 2013;20(4):e3–10. Epub 2013/03/16. 10.1111/jvh.12010 . 23490387

[14] Arora S, O'Brien C, Zeuzem S, Shiffman ML, Diago M, Tran A, et al Treatment of chronic hepatitis C patients with persistently normal alanine aminotransferase levels with the combination of peginterferon alpha-2a (40 kDa) plus ribavirin: impact on health-related quality of life. J Gastroenterol Hepatol. 2006;21(2):406–12. Epub 2006/03/03. JGH4059 [pii] 10.1111/j.1440-1746.2005.04059.x . 16509866

[15] Bonora E, Targher G, Alberiche M, Bonadonna RC, Saggiani F, Zenere MB, et al Homeostasis model assessment closely mirrors the glucose clamp technique in the assessment of insulin sensitivity: studies in subjects with various degrees of glucose tolerance and insulin sensitivity. Diabetes care. 2000;23(1):57–63. . 10857969 10.2337/diacare.23.1.57

[16] Kirschberg O, Schuttler C, Repp R, Schaefer S. A multiplex-PCR to identify hepatitis B virus—enotypes A-F. J Clin Virol. 2004;29(1):39–43. Epub 2003/12/17. S1386653203000842 [pii]. . 14675868 10.1016/s1386-6532(03)00084-2

[17] Knodell RG, Ishak KG, Black WC, Chen TS, Craig R, Kaplowitz N, et al Formulation and application of a numerical scoring system for assessing histological activity in asymptomatic chronic active hepatitis. Hepatology. 1981;1(5):431–5. . 7308988 10.1002/hep.1840010511

[18] Marcellin P, Gane E, Buti M, Afdhal N, Sievert W, Jacobson IM, et al Regression of cirrhosis during treatment with tenofovir disoproxil fumarate for chronic hepatitis B: a 5-year open-label follow-up study. Lancet. 2013;381(9865):468–75. Epub 2012/12/14. 10.1016/S0140-6736(12)61425-1 S0140–6736(12)61425–1 [pii]. . 23234725

[19] Chan WK, Nik Mustapha NR, Mahadeva S. Controlled attenuation parameter for the detection and quantification of hepatic steatosis in nonalcoholic fatty liver disease. Journal of gastroenterology and hepatology. 2014;29(7):1470–6. 10.1111/jgh.12557 . 24548002

[20] ter Borg F, ten Kate FJ, Cuypers HT, Leentvaar-Kuijpers A, Oosting J, Wertheim-van Dillen PM, et al A survey of liver pathology in needle biopsies from HBsAg and anti-HBe positive individuals. J Clin Pathol. 2000;53(7):541–8. 10961179 10.1136/jcp.53.7.541 PMC1731225

[21] Lok AS, McMahon BJ. Chronic hepatitis B: update 2009. Hepatology. 2009;50(3):661–2. 10.1002/hep.23190 . 19714720

[22] Seto WK, Lai CL, Ip PP, Fung J, Wong DK, Yuen JC, et al A large population histology study showing the lack of association between ALT elevation and significant fibrosis in chronic hepatitis B. PLoS One. 2012;7(2):e32622 10.1371/journal.pone.0032622 22389715 PMC3289659

[23] Park JY, Park YN, Kim DY, Paik YH, Lee KS, Moon BS, et al High prevalence of significant histology in asymptomatic chronic hepatitis B patients with genotype C and high serum HBV DNA levels. J Viral Hepat. 2008;15(8):615–21. 10.1111/j.1365-2893.2008.00989.x . 18573162

[24] Manesis EK, Papatheodoridis GV, Hadziyannis SJ. Serum HBV-DNA levels in inactive hepatitis B virus carriers. Gastroenterology. 2002;122(7):2092–3; author reply 3. . 12055617 10.1053/gast.2002.34021

[25] Yuen MF, Fong DY, Wong DK, Yuen JC, Fung J, Lai CL. Hepatitis B virus DNA levels at week 4 of lamivudine treatment predict the 5-year ideal response. Hepatology. 2007;46(6):1695–703. 10.1002/hep.21939 . 18027877

[26] European Association For The Study Of The L. EASL clinical practice guidelines: Management of chronic hepatitis B virus infection. Journal of hepatology. 2012;57(1):167–85. 10.1016/j.jhep.2012.02.010 . 22436845

[27] Andreani T, Serfaty L, Mohand D, Dernaika S, Wendum D, Chazouilleres O, et al Chronic hepatitis B virus carriers in the immunotolerant phase of infection: histologic findings and outcome. Clin Gastroenterol Hepatol. 2007;5(5):636–41. 10.1016/j.cgh.2007.01.005 . 17428739

[28] Hui CK, Zhang HY, Shek T, Yao H, Yueng YH, Leung KW, et al Disease progression in Chinese chronic hepatitis C patients with persistently normal alanine aminotransaminase levels. Aliment Pharmacol Ther. 2007;25(11):1283–92. 10.1111/j.1365-2036.2007.03318.x . 17509096

[29] Lesmana CR, Gani RA, Hasan I, Simadibrata M, Sulaiman AS, Pakasi LS, et al Significant hepatic histopathology in chronic hepatitis B patients with serum ALT less than twice ULN and high HBV-DNA levels in Indonesia. J Dig Dis. 2011;12(6):476–80. 10.1111/j.1751-2980.2011.00540.x . 22118698

[30] Martinot-Peignoux M, Boyer N, Colombat M, Akremi R, Pham BN, Ollivier S, et al Serum hepatitis B virus DNA levels and liver histology in inactive HBsAg carriers. Journal of hepatology. 2002;36(4):543–6. . 11943427 10.1016/s0168-8278(02)00004-1

[31] Zacharakis G, Koskinas J, Kotsiou S, Tzara F, Vafeiadis N, Papoutselis M, et al The role of serial measurement of serum HBV DNA levels in patients with chronic HBeAg(-) hepatitis B infection: association with liver disease progression. A prospective cohort study. Journal of hepatology. 2008;49(6):884–91. 10.1016/j.jhep.2008.06.009 . 18674840

[32] Papatheodoridis GV, Manesis EK, Manolakopoulos S, Elefsiniotis IS, Goulis J, Giannousis J, et al Is there a meaningful serum hepatitis B virus DNA cutoff level for therapeutic decisions in hepatitis B e antigen-negative chronic hepatitis B virus infection? Hepatology. 2008;48(5):1451–9. 10.1002/hep.22518 . 18924246

[33] Wong GL, Wong VW, Choi PC, Chan AW, Chim AM, Yiu KK, et al Clinical factors associated with liver stiffness in hepatitis B e antigen-positive chronic hepatitis B patients. Clinical gastroenterology and hepatology: the official clinical practice journal of the American Gastroenterological Association. 2009;7(2):227–33. 10.1016/j.cgh.2008.10.023 . 19121647

[34] Zoulim F, Mason WS. Reasons to consider earlier treatment of chronic HBV infections. Gut. 2012;61(3):333–6. 10.1136/gutjnl-2011-300937 . 22147510

[35] Tseng KC, Chen CY, Tsai HW, Chang TT, Chuang WL, Hsu PI, et al Efficacy of entecavir in chronic hepatitis B patients with persistently normal alanine aminotransferase: randomized, double-blind, placebo-controlled study. Antivir Ther. 2014 10.3851/IMP2754 . 24583931

[36] Perrillo RP, Lai CL, Liaw YF, Dienstag JL, Schiff ER, Schalm SW, et al Predictors of HBeAg loss after lamivudine treatment for chronic hepatitis B. Hepatology. 2002;36(1):186–94. 10.1053/jhep.2002.34294 . 12085364

[37] Lau GK, Piratvisuth T, Luo KX, Marcellin P, Thongsawat S, Cooksley G, et al Peginterferon Alfa-2a, lamivudine, and the combination for HBeAg-positive chronic hepatitis B. N Engl J Med. 2005;352(26):2682–95. 10.1056/NEJMoa043470 . 15987917

